# A note on genetic parameters and accuracy of estimated breeding values in honey bees

**DOI:** 10.1186/s12711-019-0510-6

**Published:** 2019-11-27

**Authors:** Evert W. Brascamp, Piter Bijma

**Affiliations:** Wageningen University & Research Animal Breeding and Genomics, PO Box 338, 6700 AH Wageningen, The Netherlands

## Abstract

**Background:**

In honey bees, observations are usually made on colonies. The phenotype of a colony is affected by the average breeding value for the worker effect of the thousands of workers in the colony (the worker group) and by the breeding value for the queen effect of the queen of the colony. Because the worker group consists of multiple individuals, interpretation of the variance components and heritabilities of phenotypes observed on the colony and of the accuracy of selection is not straightforward. The additive genetic variance among worker groups depends on the additive genetic relationship between the drone-producing queens (DPQ) that produce the drones that mate with the queen.

**Results:**

Here, we clarify how the relatedness between DPQ affects phenotypic variance, heritability and accuracy of the estimated breeding values of replacement queens. Second, we use simulation to investigate the effect of assumptions about the relatedness between DPQ in the base population on estimates of genetic parameters. Relatedness between DPQ in the base generation may differ considerably between populations because of their history.

**Conclusions:**

Our results show that estimates of (co)variance components and derived genetic parameters were seriously biased (25% too high or too low) when assumptions on the relationship between DPQ in the statistical analysis did not agree with reality.

## Background

In honey bees, observations on traits under selection such as honey yield are usually made on colonies, rather than on single individuals. A colony consists of a single queen and a group of thousands of workers. The phenotype of the colony is affected by the breeding value (BV) of the queen and by the mean BV of the worker group. The queen mates only on one occasion (or sometimes two) early in life with several haploid drones that were produced by queens in other colonies, so-called drone producing queens (DPQ). The queen is intended to head a colony that will be phenotyped. Each worker in the colony descends from the queen of the colony and from one of the drones that mated with her.

In bee breeding, selection of parents for the next generation entails selection of colonies from which replacement queens are reared. A replacement queen is essentially a newborn worker who receives special nutrition such that she develops into a queen, rather than into a worker. Because it is unknown from which drone a replacement queen descends, she represents a randomly selected worker from the colony. As a consequence, the estimated breeding value (EBV) of a replacement queen at the time of selection, i.e., before the colony she is going to head is phenotyped, is identical to the EBV of the worker group. Thus, for the selection of colonies to breed replacement queens, interest lies in the EBV of the worker group in the colony.

Because the BV of the worker group is the mean BV of many workers, interpretation of the variance components and heritabilities on phenotypes observed on the colony and the accuracy of selection is not straightforward. Interpretation of these parameters depends on the mean additive genetic relationship between the workers in the worker group, because this relationship affects variance of the mean BV of the worker group. This relationship in turn depends on the additive genetic relationship between the DPQ that produced the drones that mated to the queen of the colony. Moreover, while the EBV of a replacement queen reared from a colony is identical to the EBV of the worker group, the accuracies of the two EBV differ because the second is the accuracy of an average EBV.

The first purpose of this note is to clarify theoretically how the relatedness among DPQ affects phenotypic variance and heritability of a trait observed on the colony and how it affects the accuracy of the EBV of replacement queens. The second purpose is to investigate numerically how assumptions about the relatedness between DPQ in the base population affect the estimates of genetic parameters. Finally, we discuss practical implementation.

## Theory

Here, we first introduce the quantitative genetic model for traits recorded in honey bees. Next, we show (i) how phenotypic variance is calculated, and how this depends on the relatedness between DPQ, (ii) how heritability can be defined and interpreted, and (iii) how accuracy of the EBV of selection candidates is calculated. Finally, we use simulation to investigate the effects of assumptions about relatedness between DPQ in the base population on estimates of genetic parameters.

The phenotype of a colony, ${P}_{c}$, can be written as:

1 $${P}_{c}={\bar{A}}_{w}^{W}+{A}_{d}^{Q}+{E}_{c}$$

where superscripts indicate the worker-effects ($W$) and the queen-effects ($Q$) on the phenotypes, subscripts indicate the individuals, i.e. the worker group ($w$) and the queen ($d$, their dam). ${\bar{A}}_{w}^{W}$ is the average BV of the worker group for the worker effect, ${A}_{d}^{Q}$ is the BV of the queen for the queen effect, and ${E}_{c}$ is a non-heritable residual. See [1] for the biological interpretation of worker and queen-effects in the context of honey bees.

To estimate variance components, a numerator relationships matrix, $\mathbf{A}$, is derived from the pedigree [1]. The pedigree of a bee breeding program contains three types of ‘individuals’: groups of workers, dams (individual queens) and sires (groups of DPQ). As usual in an animal model, the elements of $\mathbf{A}$ are scaled relative to the additive genetic variance among individuals, ${\sigma }_{A}^{2}$, such that the estimated variance components refer to single individuals. In particular, the estimate for the variance of worker effect, ${\sigma }_{{A}^{W}}^{2}$, represents the variance of BV for the worker effect among single individuals, rather than among the groups of workers, ${\sigma }_{{\bar{A}}_{w}^{W}}^{2}$.

### Phenotypic variance

It follows from Eq. (1) that the phenotypic variance equals:

2 $${\sigma }_{P}^{2}={\sigma }_{{\bar{A}}_{w}^{W}}^{2}+{\sigma }_{{A}_{d}^{Q}}^{2}+2{\sigma }_{{\bar{A}}_{w}^{W}{A}_{d}^{Q}}+{\sigma }_{{E}_{c}}^{2},$$

where ${\sigma }_{{\bar{A}}_{w}^{W}}^{2}$ is the additive genetic variance for the worker effect of worker groups, ${\sigma }_{{A}_{d}^{Q}}^{2}$ the additive genetic variance for the queen effect of dams, and ${\sigma }_{{\bar{A}}_{w}^{W}{A}_{d}^{Q}}$ their covariance.

Worker groups consists of many ($n$) related workers, such that the relationship between ${\sigma }_{{A}^{W}}^{2}$ and ${\sigma }_{{\bar{A}}_{{w}_{i}}^{W}}^{2}$ for worker group $i$ is given by

3 $${\sigma }_{{\bar{A}}_{{w}_{i}}^{W}}^{2}=\frac{1+\left(n-1\right){a}_{ii}}{n}{\sigma }_{{A}^{W}}^{2}={a}_{ii}{\sigma }_{{A}^{W}}^{2},$$

where ${a}_{ii}$ is the additive genetic relationship between two random workers from the same colony, and depends on the distribution of drone contributions to the worker group (see “Methods” section and Brascamp and Bijma [1] for details). The second equality holds because $n$ is very large.

To find ${a}_{ii}$, consider the typical structure of a bee breeding programme where the workers in a colony are related not only because they descend from the same dam, but also because they either descend from the same drone, from two drones of the same DPQ, or from two different DPQ reared from the same colony. From Eq. 24 of [1], the average additive genetic relationship between workers in a colony of a non-inbred population equals:

4 $${a}_{ii}=\frac{1}{4}+\frac{1}{2}{p}_{1}+\frac{1}{4} ({p}_{2}-{p}_{1})+\frac{1}{4}\left(1-{p}_{2}\right){a}_{ss},$$

where the first term is relatedness due to the shared mother, ${p}_{1}$ is the probability that two workers descend from the same drone, ${p}_{2}$ is the probability that they descend from the same DPQ, and ${a}_{ss}$ is the additive genetic relationship between two DPQ reared from the same colony.

If the DPQ in the base population are unrelated, Eq. (4) reduces to:

5u $${a}_{{base}_{u}}=\frac{1}{4}+\frac{1}{4}{p}_{1}+\frac{1}{4}{p}_{2},$$

where subscript $u$ indicates “unrelated”. However, within a few generations the ${a}_{ss}$ increases to an equilibrium value such that ${a}_{ii}={a}_{ss}$ [2]. In other words, about four generations after the base generation, the ${a}_{ss}$ has approached an equilibrium value, while the overall population is still unrelated and non-inbred. Thus, this equilibrium value may already be present in the base population, if the base population was part of an already ongoing breeding program. In that case, ${a}_{ii}={a}_{ss}$, such that the equilibrium value becomes:

5e $${a}_{{base}_{e}}=\frac{1+{p}_{1}+{p}_{2}}{3+{p}_{2}},$$

where subscript $e$ indicates “equilibrium”. Apart from the gradual increase in inbreeding on the population level, the value of ${a}_{ss}$ remains at its equilibrium thereafter.

### Heritability

A common way to define heritability (${h}^{2}$) is as the fraction of phenotypic variance that can be attributed to additive genetic effects in the base population [3]. From Eqs. (2) and (3), overall heritability and heritabilities of the worker and queen effects are:

6a $${h}_{V}^{2}=\frac{{a}_{base}{\sigma }_{{A}^{W}}^{2}+{\sigma }_{{A}^{W}{A}^{Q}}+{\sigma }_{{A}^{Q}}^{2}}{{\sigma }_{P}^{2}},$$

6b $${h}_{{W}_{V}}^{2}=\frac{{a}_{base}{\sigma }_{{A}^{W}}^{2}}{{\sigma }_{P}^{2}},$$

6c $${h}_{{Q}_{V}}^{2}=\frac{{\sigma }_{{A}^{Q}}^{2}}{{\sigma }_{P}^{2}},$$

where the subscript $V$ indicates heritabilities defined as a fraction of the variance, and either the unrelated (5u) or equilibrium (5e) value of ${a}_{base}$ should be used in the numerator and in ${\sigma }_{P}^{2}$ depending on whether DPQ in the base population are unrelated or have an equilibrium relatedness. Note that also ${\sigma }_{P}^{2}$ depends upon ${a}_{base}$ (Eqs. (2) and (3)). The middle term in the numerator of Eq. (6a) uses a relationship of ½ between the queen and her workers.

When traits are affected by genetic effects that originate from different individuals, for example with direct and maternal effects, direct and social effects, or worker and queen effects, defining heritability as the fraction of phenotypic variance attributable to additive genetic effects does not reflect the potential of a population to respond to selection [4, 5]. Here, this issue is relevant for two reasons; first, the phenotype of a colony depends on both the queen and the workers, and second, the group of workers consists of multiple individuals. Because response to selection depends on the sum of changes in the mean worker and mean queen effects, based on [5, 6] the relevant “heritability” for response to selection is:

7a $${T}^{2}=\frac{{\sigma }_{{A}^{W}}^{2}+2{\sigma }_{{A}^{W}{A}^{Q}}+{\sigma }_{{A}^{Q}}^{2}}{{\sigma }_{P}^{2}}.$$

Analogously, we may express the total heritable variance for response to selection due to workers and queens relative to phenotypic variance:

7b $${T}_{W}^{2}=\frac{{\sigma }_{{A}^{W}}^{2}}{{\sigma }_{P}^{2}},$$

7c $${T}_{Q}^{2}=\frac{{\sigma }_{{A}^{Q}}^{2}}{{\sigma }_{P}^{2}}.$$

These results are a special case of the general approach of [5]. ${T}_{Q}^{2}$ is equal to the ordinary heritability of queen effects, because the queen is a single individual. Note that theoretically ${T}^{2}$ and ${T}_{W}^{2}$ can exceed 1, as the denominator, ${\sigma }_{P}^{2}$, contains ${\sigma }_{{\bar{A}}^{W}}^{2}$ while the numerator contains ${\sigma }_{{A}^{W}}^{2}$.

### Accuracy of EBV

Fitting Eq. (1) yields the best linear unbiased prediction (BLUP) of the mean BV of worker groups, $\widehat{{\bar{A}}_{w}}$, and corresponding accuracies, say ${r}_{w}$. Interest, however, also lies in the accuracy, ${r}_{q}$, of the EBV of a replacement queen bred from the colony. The EBV of the replacement queen, $\widehat{{A}_{q}}$, equals the EBV of the worker group:

8 $$\widehat{{A}_{q}}=\widehat{{\bar{A}}_{w}}.$$

Note that we dropped the superscript for the trait (workers vs. queen effect), because this section applies equally to both traits. In the section entitled “Implementation”, we show how the accuracy of the sum of worker and queen effect can be calculated.

In general, the accuracy of BLUP equals the ratio of the standard deviations of EBV and BV (e.g. [7]), such that:

9 $${r}_{q}=\frac{{\sigma }_{\widehat{{A}_{q}}}}{{\sigma }_{{A}_{q}}}=\frac{{\sigma }_{\widehat{{\bar{A}}_{w}}}}{{\sigma }_{{A}_{q}}},$$

where the second step follows from Eq. (8). Using ${\sigma }_{\widehat{{\bar{A}}_{w}}}={r}_{w}{\sigma }_{{\bar{A}}_{w}}$ and ${\sigma }_{{\bar{A}}_{w}}=\sqrt{{a}_{ii}}{\sigma }_{{A}_{w}}$ from Eq. (3), and ${\sigma }_{{A}_{w}}={{\sigma }_{A}}_{q}$, it follows that:

10 $${r}_{q}=\sqrt{{a}_{ii}}{r}_{w}.$$

This result shows that the accuracy of the EBV for a replacement queen is a factor $\sqrt{{a}_{ii}}$ smaller than the accuracy of the EBV for the worker group. Thus, although the EBV of the worker group and of a replacement queen are identical (Eq. (8)), accuracy of the EBV is lower for the replacement queen (Eq. (10)), because the genetic variance among replacement queens is larger than that among worker groups. The size of ${a}_{ii}$ follows from Eq. (4) in the course of developing $\mathbf{A}$ according to [1].

The value for ${r}_{w}$ follows from the standard error of prediction (SEP) of the BLUP in the usual way,

11 $${r}_{w}=\sqrt{1- \frac{{SEP}_{w}^{2}}{{\sigma }_{{\bar{A}}_{w}}^{2}}}.$$

Note that the denominator of the second term in the square root is ${\sigma }_{{\bar{A}}_{w}}^{2}$ and not ${\sigma }_{{A}_{w}}^{2}$.

## Methods

### Base population history

Equations (5u) and (5e) show that the relationship between DPQ in the base population (${a}_{base}$) depends on the population’s history. When the base population is part of an ongoing bee breeding program, this relationship is higher (Eq. (5e)) than for a newly started breeding program (Eq. (5u)).

### Simulation

To study the effect of erroneous assumptions for ${a}_{base}$ on estimates of genetic parameters, we carried out simulations. For both the simulations and the analysis of the simulated data, we considered two alternatives for ${a}_{base}$, either unrelatedness (Eq. (5u)) or equilibrium values (Eq. (5e)). Thus, we investigated a 2 × 2 design, with a total of four scenarios. Each scenario was replicated 40 times. The equations needed for the simulation were taken from [1].

In the first year, DPQ (sires) were simulated that produce drones fertilizing queens that were simulated in the second year. Sires in the first three years and dams in years two and three were base parents. Base dams were unrelated. The additive genetic relationships between DPQ in base sires either corresponded to Eqs. (5u) or (5e). We assumed that ${p}_{1}$ and ${p}_{2}$ followed from a Poisson distribution, so that ${p}_{1}=\frac{1}{D}$ and ${p}_{2}=\frac{1}{D}+\frac{1}{S}$ [1]. Each queen in average mated with 12 drones ($D=12$) that descended from 10 different sires ($S=10$). With these values, relationships between DPQ in the base population were ${a}_{{base}_{u}}=0.32,$ and ${a}_{{base}_{e}}=0.40.$

Every year, 10 sires ($S$) were mated to 10 queens each, and each queen produced five offspring. This led to 500 queens with colonies and phenotypes per year. Queens belonging to the same full-sib family were mated to the same sire. Sires were assigned at random, with the restriction that each sire contributed 10 $\times$ 5 = 50 offspring. Every year one new sire was selected at random from each sire family (1 out of 50), and one new dam was selected at random from each dam family (1 out of 5). Simulated genetic parameters were  ${\sigma }_{{A}^{W}}^{2}=1$,  ${\sigma }_{{A}^{Q}}^{2}=0.5$, the genetic correlation between worker and queen effect was − 0.5, and  ${\sigma }_{{E}_{c}}^{2}=2$.

For all datasets, variance components were estimated with ASReml [8], using a relationship matrix $$\mathbf{A}$$ that either assumed unrelated DPQ in the base population ($${a}_{ii}=0.32)$$, or equilibrium relationships between DPQ ($${a}_{ii}=0.40)$$. Inputs for ASReml were identifications of groups of workers and dams, the observations, and the inverse of the relationship matrix according to [1].

## Results

Results in Table 1 show that the estimates of the (co)variance components and derived parameters were unbiased when the assumption for the base sires in the analysis agreed with the simulation. When the simulation assumed unrelated base animals while the analysis assumed equilibrium relationships, the variances of worker and queen effects and their covariance were underestimated by a factor of approximately $\frac{{a}_{{base}_{u}}}{{a}_{{base}_{e}}}=\frac{0.32}{0.40}=0.8$. Heritabilities were also underestimated. When the simulation assumed equilibrium relationships while the analysis assumed unrelated base animals, results mirrored the previous situation and the (co)variance components were overestimated by a factor of approximately $\frac{0.40}{0.32}=1.25$.

**Table 1:** Estimates of (co)variance components (and standard errors) resulting from the simulation study and analysis assuming base populations with unrelated or equilibrium relationships among DPQ

Parameters^a^	Simulation unrelated			Simulation equilibrium		
	True value	Analysis unrelated	Analysis equilibrium	True value	Analysis unrelated	Analysis equilibrium
${\sigma }_{{A}^{W}}^{2}$	1.00	1.01 (0.04)	0.72 (0.03)	1.00	1.44 (0.07)	0.95 (0.04)
${\sigma }_{{A}^{Q}}^{2}$	0.50	0.50 (0.02)	0.42 (0.02)	0.50	0.59 (0.03)	0.48 (0.02)
$cov\left({A}^{W}{A}^{Q}\right)$	− 0.35	− 0.36 (0.03)	− 0.23 (0.02)	− 0.35	−0.51 (0.03)	− 0.30 (0.02)
${\sigma }_{E}^{2}$	2.00	2.01 (0.01)	2.05 (0.01)	2.00	1.95 (0.01)	2.00 (0.01)
${\sigma }_{{\bar{A}}^{W}}^{2}$	0.32	0.32 (0.01)	0.29 (0.01)	0.40	0.46 (0.02)	0.38 (0.02)
${\sigma }_{P}^{2}$	2.47	2.47 (0.01)	2.53 (0.01)	2.55	2.49 (0.01)	2.56 (0.01)
${h}_{{W}_{V}}^{2}$	0.13	0.13 (0.01)	0.11 (0.00)	0.16	0.18 (0.01)	0.15 (0.01)
${T}_{W}^{2}$	0.41	0.41 (0.02)	0.28 (0.01)	0.39	0.58 (0.03)	0.37 (0.01)
${T}_{Q}^{2}$	0.20	0.20 (0.01)	0.17 (0.01)	0.20	0.24 (0.01)	0.19 (0.01)
${T}^{2}$	0.32	0.32 (0.01)	0.27 (0.01)	0.31	0.40 (0.02)	0.32 (0.01)
${r}_{g}$	− 0.50	− 0.49 (0.03)	− 0.41 (0.03)	− 0.50	− 0.54 (0.02)	− 0.44 (0.02)

^a^See text

## Implementation

### Genetic parameters

For practical implementation, it is important to have a good understanding of the additive genetic relationship between DPQ in the base generation. For a starting breeding programme, these queens can be considered as unrelated, as assumed for example in [9]. If the colonies with these queens are kept at mating stations similar to the breeding programme that is starting up, then the relationship between DPQ may be calculated assuming that the number of DPQ equals the usual number in the programme. If there is open mating in the base population, a far larger number of DPQ should be used in Eqs. (5). See Eqs. (24a) and (24b) in [1] for how the number of DPQ affects ${a}_{base}$. Baudry et al. [10] reported a value of 240 DPQ with open mating, but this number will obviously depend on the concentration of colonies and the geography of the region. When the analysis concerns a dataset that involves recent years in a longer-running programme, a reasonable assumption is that the additive genetic relationship between DPQ is in equilibrium.

### Accuracy

If the interest lies in the BV for queen effect, or in the BV for worker effect, but not in their sum, then the SEP required to calculate the accuracy of the EBV is usually included in the output of software for breeding value estimation (*e.g.*, ASReml [8]). However, when the genetic analysis includes both worker and queen effects, the interest usually lies in the summed EBV for worker and queen effects of replacement queens, ${\widehat{A}}_{q}^{W}+{\widehat{A}}_{q}^{Q}$, because this “index” determines response to selection. To calculate the accuracy of this index for a prospective replacement queen, we first need the SEP of the corresponding value for the worker group of the colony, ${\widehat{\bar{A}}}_{w}^{W}+{\widehat{\bar{A}}}_{w}^{Q}$. The SEP of the index for the worker group depends on the diagonals of the inverse of the coefficient matrix for queen and worker effect of the worker group and on the corresponding off-diagonal. In ASReml, the $SEP$ of the worker group can be obtained by inclusion of the command line (personal communication Arthur Gilmour)

$$\mathrm{PREDICT}\; \mathrm{worker}\;\mathrm{a}{:}\mathrm{b}\; \mathrm{queen}\; \mathrm{a}{:}\mathrm{b}\,\, !$$

12 $$\mathrm{ONLY}\;\mathrm{USE}\; \mathrm{worker\,\,queen\,\,!}\; \mathrm{PARALLEL}\; \mathrm{worker\,\,queen}$$

directly after the line defining the model. In Eq. (12), $\mathrm{a}$ and $\mathrm{b}$ refer to sequence numbers in the pedigree file (of note: we experienced a limit to the size of $\mathrm{b}-\mathrm{a}$ that can be dealt with in a single ASReml-run.) Subsequently, the accuracy of the index for a replacement queen follows from substituting the SEP for the worker group into Eq. (11), where ${\sigma }_{{\bar{A}}_{w}}^{2}$ now refers to the sum of both BV, ${\sigma }_{{\bar{A}}_{w}}^{2}={a}_{ii}({\sigma }_{{A}^{W}}^{2}+2{\sigma }_{{A}^{W}{A}^{Q}}+{\sigma }_{{A}^{Q}}^{2})$, and then applying Eq. (10).

## Conclusions

Our results show that estimates of (co)variance components and derived genetic parameters are seriously biased (25% too high or too low) when assumptions in the statistical analysis on the relationship between DPQ in the base population do not agree with reality. Generally, the history of the base population will be sufficiently well known to decide what assumption to make such that the bias can be avoided.

## References

[1] Brascamp EW, Bijma P (2014). Methods to estimate breeding values in honey bees. Genet Sel Evol..

[2] Bienefeld K, Pirchner F (1990). Heritabilities for several colony traits in the honeybee (*Apis mellifera carnica*). Apidologie.

[3] Falconer DS, Mackay TFC (1996). Introduction to quantitative genetics.

[4] Willham RL (1976). The covariance between relatives for characters composed of components contributed by related individuals. Biometrics.

[5] Bijma P (2011). A general definition of the heritable variation that determines the potential of a population. Genetics.

[6] Bergsma R, Kanis E, Knol EF, Bijma P (2008). The contribution of social effects to heritable variation in finishing traits of domestic pigs (*Sus scrofa*). Genetics.

[7] Mrode RA (2014). Linear models for the prediction of animal breeding values.

[8] Gogel BJ, Gilmour AR, Welham SJ, Cullis BR, Thompson R (2015). ASReml Update: What’s new in Release 4.1.

[9] Brascamp EW, Willam A, Boigenzahn C, Bijma P, Veerkamp RF (2018). Correction to: Heritabilities and genetic correlations for honey yield, gentleness, calmness and swarming behaviour in Austrian honey bees. Apidologie.

[10] Baudry E, Solignac M, Garnery L, Gries M, Cornuet JM, Koeniger N (1998). Relatedness among honeybees (*Apis mellifera*) of a drone congregation. Proc Biol Sci..

